# Insecticides Susceptibility Status of the Bedbugs (*Cimex lectularius*) in a Rural Area of Magugu, Northern Tanzania

**DOI:** 10.4103/0974-777X.56252

**Published:** 2009

**Authors:** Eliningaya J Kweka, Beda J Mwang'onde, Epiphania E Kimaro, Shandala Msangi, Filemoni Tenu, Aneth M Mahande

**Affiliations:** *Tropical Pesticides Research Institute, Division of Livestock and Human Diseases Vector Control, Arusha, Tanzania*; 1 *National Institute for Medical Research, Amani Research Station, Muheza, Tanga, Tanzania*

**Keywords:** Bedbugs, Insecticides, Magugu, Pyrethroids, Susceptibility status, Tanzania

## Abstract

The recent spread of bedbugs, *Cimex lectularius L.* (Heteroptera: Cimicidae), has received attention of the public health sector for designing of effective plan of action for control. Several studies have focused on determining the distribution and abundance of bedbug populations in tropical areas. This study establishes baseline information on deltamethrin, permethrin, alphacypermethrin, lambdacypermethrin and K-O tab susceptibility status in a bedbug population collected from Magugu area in northern Tanzania. The evolution of insecticide resistance could be a primary factor in explaining this resurgence of bedbugs in many areas, both rural and urban. Evaluation of the bedbug population from houses in Magugu indicates that the population of bedbugs is susceptible to pyrethroid insecticides, which are commonly used. Without the development of new tactics for bedbug resistance management, further escalation of this public health problem should be expected when resistant gene spreads within the population. These results suggest that although all concentrations kill bedbugs, more evaluations should be done using WHO kits and mechanisms involved in pyrethroid resistance should be evaluated, such as metabolic and knockdown resistance gene, to have a broad picture for better design of control methodologies.

## INTRODUCTION

The common tropical bedbug *(Cimex lectularius)* is a dorsoventrally flat, wingless, hematophagous insect.[1] It is found in most parts of the world, and infestations are common where the house design is poor and living conditions are unsanitary, even today.[2] The poor hygienic conditions of shelters have attributed to high infestations of bedbugs in most areas.[3] Bedbugs strictly feed on warm-blooded animals.[4–7] They suck blood, causing discomfort and allergic skin reactions due to their salivary proteins.[4,5,8,9] *Cimex hemipterus* have not been reported to be involved in disease transmission.[9] These bedbugs, however, have been shown to harbor human immunodeficiency virus (HIV).[10] Some studies have shown that the common bedbug can carry hepatitis B virus (HBV) for up to 5 weeks after the uptake of infectious blood meal,[11] suggesting that biological or mechanical transmission of HBV may be possible[12]; but other studies done in Gambia, West Africa, have not shown decrease of hepatitis B transmission despite sharp decline in the population of bedbugs.[13] It is evident that heavy bedbug infestations can lead to iron and hemoglobin deficiencies in humans, mostly in children, and cause loss of sleep.[14,15] Currently there is neither a trap nor an odor-baited device for sampling bedbugs.[16] Traps used to sample other insects have proved to be inefficient or unreliable for bedbugs.[4] Therefore, the use of insecticides for bedbugs is still the most promising and reliable method of control.[15,16] Insect genera members have been reported to show resistance to different insecticides.[17,18] Several parts of Africa have been reported to show tolerance of bedbugs to pyrethroids used for bed nets and indoor residual sprays,[19–22] which is an impact of intensified use of these insecticides.

In Tanzania, there have been observations of bedbug resistance to pyrethroids from areas where there has been massive promotion of the use of bed nets, in Bagamoyo district in coastal region[23] and in Muheza district in Tanga region.[24] Currently in Tanzania, there is a national scaling-up program for insecticides treated bed nets (ITNs) coverage as a major strategy for malaria vectors control.[23] Due to this campaign, ITNs distributions and their large coverage in Tanzania since 2005 might be the source of causing resistant among bedbugs as observed in coastal and Tanga regions. The insecticides susceptibility status of bedbugs at Magugu ward has not been evaluated, and the area has moderate malaria transmission and is one of the areas prone to malaria epidemics in Tanzania, hence the high bed net coverage.[25] The investigation reported here was undertaken in order to provide the baseline information on bedbug susceptibility and the status of resistance to pyrethroid insecticides.

## MATERIAL AND METHODS

### Study area

The study was conducted at Magugu (4′ 00 S, 35′ 46 E, 913 m above sea level) in Babati district in Manyara region of northern Tanzania. The area is occupied by three major tribes (Iraqw, Mang'ati and Mbugwe); and minorities are Chaga, Nyaturu, Pare and Nyiramba. The area is highly active in agriculture. The houses are still constructed in the traditional style, with improvement in peri-urban areas.

### Ethical consideration

Ethical and scientific approval was obtained from the research ethics committee of the Tropical Pesticides Research Institute at Arusha, Tanzania. Before house selection, all villagers were invited to attend the meeting which elaborated the essence of the study to that community. After thorough explanation, all people who had bedbug infestation in their homes were selected for the study. Shelter owners and residents gave written informed consent before bedbug collection.

### Insecticide dosages preparation and surface treatments

Deltamethrin (supplied by Coopers Limited, 99% purity), permethrin (supplied by Coopers Limited, Australia, purity 99%), alphacypermethrin (supplied by Norbrook Laboratories Limited, 95.7% purity) and lambdacypermethrin (supplied by Coopers Limited, Australia, 97.0% purity) were used. Filter paper disks (9.0 cm diameter, Whatman no. 1) were dipped for 10 seconds into a required concentration (in parts per million) of deltamethrin, permethrin, alphacypermethrin and lambdacyalothrin solution in acetone. Disks were air-dried in a fume hood for 20-30 min, wrapped with aluminum foil, placed into a plastic bag and stored at 20°C until used. The concentration was converted to ‘parts per million’ (ppm). The conversion was done on the basis of 1 ppm being equal to 1 mg per liter or 0.001g per liter. This means that if preparation of 200 ppm was needed from 97% purity technical material, 0.2 g was equated to 97% and then 100% equivalent was calculated [(100×0.2)/97= 0.206]. The 0.206 g of 97% purity technical material dissolved in 1 liter of distilled water produced a concentration of 200 ppm of that technical material. For concentration dilution, Fletcher and Axtell[26] methodology was used, which is known to be consistent. The concentration was prepared by using the purity grade of the technical materials as described by Fletcher and Axtell.[26] The piece of netting material was analyzed for amount of water enough to wet it. Then, the amount of insecticide was diluted in the same amount of water for wetting the netting material (the method of conventional treatment of bed net), as described by Sahu *et al.*[27]

In this experiment, a net impregnated with K-O tabs was evaluated as described by Myamba *et al.*[23] and by Temu *et al.*[24]

### Bedbug collection and resistance status evaluation

The blood-fed bedbugs were collected from the houses of residents, with informed consent. Collection technique used was as described by Newberry and Jansen.[28] Two senior field entomology technicians collected the bedbugs, from 7 am to 8 am, from the rooms where people spent the night everyday. The bedbugs were collected from the bedding and bed frames, cracks in walls and all other possible harborages. The bedbugs were sorted in a laboratory to separate nymphs and adults. Nymphs were killed in charcoal fire. The houses were made up of mud or brick walls and corrugated iron or thatched roofs. Fifteen houses were selected under three categories: houses with bed nets, without bed nets and with torn bed nets. The concentration-response data were collected by exposing blood-fed adult bedbugs to different concentrations of each chemical (technical material dissolved in distilled water based on wt.: vol. ratio) on netting material of filter paper with a diameter of 9.0 cm on inverted Petri dish. Netting material was treated with 0.9 mL of dilutions of insecticides. Concentrations were expressed in ‘parts per million’ of active ingredient. Six concentrations from each insecticide causing mortality from 5% to 95% were selected on the basis of screening described by Fletcher and Axtell.[26] A replica consisted of treated filter paper with 10 bedbugs and four replicas per dilution on 4 different days.[26] Mortality was determined after 24 hours and 48 hours by counting the bedbugs that did not move when the dish was tapped.

### Statistical analysis

Data were entered in MS-Excel for validation. Analysis was performed with SPSS version 15.0 for Windows, and regression probit analysis program was used to assess the dosage response in all replicas of each insecticide. LD_50,_ LD_95_ and 95% confidence interval were calculated for 24- and 48-hour mortalities.

## RESULTS

**Figure 1:**
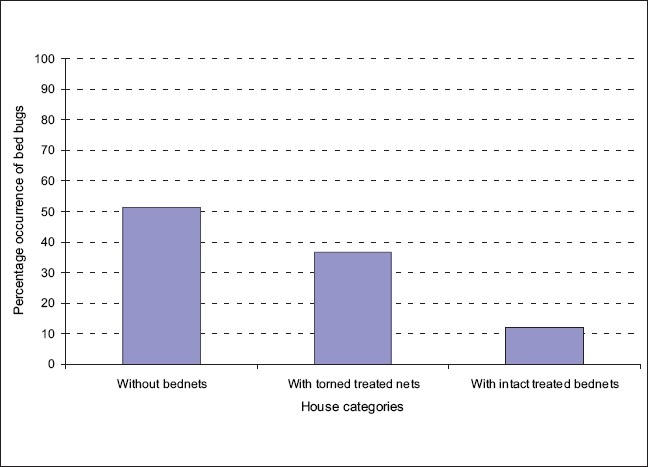
Distribution of adult bedbugs in houses with intact, torn and untreated bed nets

A total of 3676 bedbugs were collected, of which 2666 bedbugs were adults and 1010 were nymphs. Among the adults, 1370 (51.3%) bedbugs were collected from houses with no bed nets, 980 (36.8%) bedbugs were collected from houses with torn bed nets and 316 (11.9%) were collected from houses with intact treated bed nets. Out of the 2666 bedbugs, 1500 were used in experiments after being observed to be fit for susceptibility test. As many as 1166 bedbugs had deformities, which included leg loss during collection. The abundance of bedbugs in each house category is shown in Figure 1. The results with regard to susceptibility status are shown in Table 1. There was no immediate mortality other than that after 24 hours and 48 hours of exposure to insecticides. The 50% and 95% confidence intervals in a population of bedbugs in the 24 hours and 48 hours after exposure to insecticides (in dose response experiments) are shown in Table 1.

**Table 1:** The mean dosage to cause 50% to 95% mortality in the 24 hours and 48 hours after exposure of bed bugs to insecticides

Time	Treatment	LD_50_		LD_95_	
			
		Mean dosage	95% CI	Mean dosage	95% CI
24 hours	Deltamethrin	54.4	22.3 – 84.8	500.2	473.3 – 527.1
	Alphacypermethrin	10.7	8.3 – 13.2	164.3	137.5 – 191.1
	KO-Tab	61.7	49.1 – 74.3	520.3	465.8 – 574.8
	lambdacyalothrin	115	84.2 – 113.2	563.6	498.9 – 628.3
	Permethrin	111.8	91.2 – 111.8	369.1	301.4 – 436.8
48 hours	Deltamethrin	10.3	7.1 – 19.5	65.2	49.3 – 81.1
	Alphacypermethrin	13.9	11.5 – 16.5	49.2	37.2 – 63.5
	KO-Tab	17.9	12.3 – 22.8	82.5	60.2 – 107.5
	lambdacyalothrin	17.5	12.5 – 21.6	62.9	47.3 – 78.5
	Permethrin	6.1	3.03 – 10.1	26.2	18.3 – 36.4

## DISCUSSION

The findings in these series of experiments have shown that common pyrethroids used in bed net treatment are still the most efficient insecticides in the control of bedbugs, as is also shown in other studies.[18] Therefore, proper use of ITNs and long lasting insecticides treated bed nets (LLITNs) against mosquitoes can play a major role in reducing the bedbug population by increasing the rates of their mortality based on their observed susceptibilities to the pyrethroids used.[23,24] The observations from this study can have high impact on the recommendations to add value to the scaling-up programs for ITN and LLITNs, whose introduction in some rural villages of Tanzania has shown rapid decline in infestation by bedbugs in these areas of Tanzania.[23,24] The observed results are promising and valuable in scaling up the ITNs, with the awareness of not using a single insecticide but rather using a combination for reducing the chance of resistance buildup in insects.[29] In Gambian houses, introduction of bed nets resulted in a massive reduction of indoor arthropods, including bedbugs.[23,24] The pyrethrum dosage used to impregnate bed nets is higher than the concentrations that were used in these experiments. The recommended concentrations of pyrethroids for indoor residual spray by WHO are permethrin (0.2-0.5 g/m^2^), deltamethrin (0.02-0.025 g/m^2^) and alphacypermethrin (0.02-0.03 g/m^2^).[30] Using bed nets in African countries, particularly in rural Tanzania, is better than using indoor residual sprays, due to high frequency of re-plastering of house walls.[31] It is therefore important to continue scaling up the ITNs for use in rural areas, where bedbug infestations are higher, rather than using indoor residue spray (IRS). In this study, infestation has been observed to be mostly in houses without bed nets and in houses with untreated bed nets [Figure 1]. In terms of the cost of re-treatment and follow-up, the use of LLITNs with subsidized value is recommended.[32] The voucher scheme for pregnant women in Tanzania (commonly known as Hati Punguzo) to get subsidized bed nets has increased mosquito net coverage in Tanzania, hence more protection for mother and child against mosquitoes and bedbugs is achieved[33]; though further scaling up and subsidizing the net costs for all groups are important to maximize the coverage for both malaria vectors control and other arthropods.[34] Appropriate training on control of bedbugs and other domestic pests should be given to community health trainers.[34] The proper use of ITNs and LLITNs will discourage indoor harboring of bedbugs and other domestic pests,[35] as all bedbugs evaluated were 100% susceptible to the insecticides used. More sectors should be involved in this scaling up and subsidization of the ITNs and LLITNs so as to increase the coverage, which is low in rural areas of Tanzania.[36]

## CONCLUSION

It can be concluded from the findings of this study that pyrethroids-impregnated bed nets can contribute to the eradication of bedbugs in infested houses of Magugu area, but more studies have to be done using standardized diagnostic dosages recommended by WHO. Properly treated bed nets, partially treated beds and bedding material can be used to increase bedbug mortality. Continuous assessment of bedbug susceptibility status in areas with broad bed net coverage is also of paramount.
